# ALOX15-launched PUFA-phospholipids peroxidation increases the susceptibility of ferroptosis in ischemia-induced myocardial damage

**DOI:** 10.1038/s41392-022-01090-z

**Published:** 2022-08-15

**Authors:** Xiao-Hui Ma, Jiang-Han-Zi Liu, Chun-Yu Liu, Wan-Yang Sun, Wen-Jun Duan, Guan Wang, Hiroshi Kurihara, Rong-Rong He, Yi-Fang Li, Yang Chen, Hongcai Shang

**Affiliations:** 1 Guangdong Engineering Research Center of Chinese Medicine & Disease Susceptibility, Jinan University, Guangzhou, 510632 China; 2 Guangdong Province Key Laboratory of Pharmacodynamic Constituents of TCM and New Drugs Research, College of Pharmacy, Jinan University, Guangzhou, 510632 China; 3 International Cooperative Laboratory of Traditional Chinese Medicine Modernization and Innovative Drug Development of Chinese Ministry of Education (MOE), Jinan University, Guangzhou, 510632 China; 4 Institute of Traditional Chinese Medicine, Xinjiang Medical University, Urumqi, 830054 China; 5 Innovation Center of Nursing Research, Nursing Key Laboratory of Sichuan Province, State Key Laboratory of Biotherapy and Cancer Center, West China Hospital, Sichuan University, Chengdu, 610041 China; 6 College of Pharmacy, Guangzhou University of Chinese Medicine, Guangzhou, 510405 China; 7 Key Laboratory of Chinese Internal Medicine of Ministry of Education, Dongzhimen Hospital, Beijing University of Chinese Medicine, 100700 Beijing, China

**Keywords:** Cardiology, Diseases, Cell biology

## Abstract

Myocardial ischemia/reperfusion (I/R) injury is a classic type of cardiovascular disease characterized by injury to cardiomyocytes leading to various forms of cell death. It is believed that irreversible myocardial damage resulted from I/R occurs due to oxidative stress evoked during the reperfusion phase. Here we demonstrate that ischemia triggers a specific redox reaction of polyunsaturated fatty acids (PUFA)-phospholipids in myocardial cells, which acts as a priming signaling that initiates the outbreak of robust oxidative damage in the reperfusion phase. Using animal and in vitro models, the crucial lipid species in I/R injury were identified to be oxidized PUFAs enriched phosphatidylethanolamines. Using multi-omics, arachidonic acid 15-lipoxygenase-1 (ALOX15) was identified as the primary mediator of ischemia-provoked phospholipid peroxidation, which was further confirmed using chemogenetic approaches. Collectively, our results reveal that ALOX15 induction in the ischemia phase acts as a “burning point” to ignite phospholipid oxidization into ferroptotic signals. This finding characterizes a novel molecular mechanism for myocardial ischemia injury and offers a potential therapeutic target for early intervention of I/R injury.

## Introduction

Ischemia/reperfusion (I/R) injury, the leading cause of myocardial damage,^1–4^ is characterized by an inflammatory response that causes further damage to viable tissue around the infarct. Ischemia (blood flow restriction) and reperfusion (blood flow restoration) are two main phases of I/R injury. It has been widely accepted that the reperfusion stage of I/R rather than the ischemia stage is responsible for causing irreparable pathophysiological consequences to myocardial cells through an increase in oxygen free radicals that trigger oxidative stress induced cellular death.^5^ This long held belief was based on the understanding that reperfusion results in the sudden re-entry of oxygen,^5,6^ leading to exposure of the tissue to reactive oxygen species (ROS) including superoxide anion (O^2•‒^), hydrogen peroxide (H_2_O_2_) and hydroxyl radical (HO^•^) among others. However, more recent studies have suggested that reperfusion-induced oxidative injury is dependent on critical events such as succinate accumulation^7,8^ occurring during the ischemia stage, suggesting the necessity of taking deep insight into ischemia-related molecular changes. Indeed, cardioprotective interventions targeting ischemic episodes are efficient to reduce morbidity and mortality in I/R.^9,10^ Nonetheless, the intrinsic molecular determinants of ischemia injury, especially those related to oxidative stress, remain largely unknown.

In recent years, phospholipid peroxidation, a lipoxygenase-mediated specific oxidative response, has attracted much attention due to its critical role as the final executor of ferroptosis in many diseases.^11,12^ Ferroptosis has now been appreciated as a type of oxidative cell death with distinct properties and functions, and is prominently characterized by the accumulation of lethal lipid ROS which is initiated by the peroxidation of phospholipids having polyunsaturated fatty acid (PUFA) chains.^12,13^ Till now, several studies have suggested a role for ferroptosis in myocardial damage during reperfusion.^14–16^ Nevertheless, these studies ignored the presence of phospholipid peroxidation and ferroptosis in ischemia.

In the present study, we propose that a disturbance of PUFA-phospholipids launch pre-oxidative conditions during the ischemia phase. This provides a priming signal for an outbreak of robust oxidative damage in the reperfusion phase. Using PUFA-enriched cells and animals we show that the lipoxygenase, arachidonic acid 15-lipoxygenase-1 (ALOX15) initiates specific redox reactions in PUFA-phospholipids, which increases susceptibility to ischemia injury. We also identified the efficacy of daidzein, a natural cardioprotective molecule to target ALOX15. Our findings provide new insights into the pathogenic mechanisms of myocardial ischemia injury and identify potential therapeutic targets for early treatment of I/R.

## Results

### Phospholipid peroxidation is triggered by hypoxia in cardiomyocytes

**Fig. 1:**
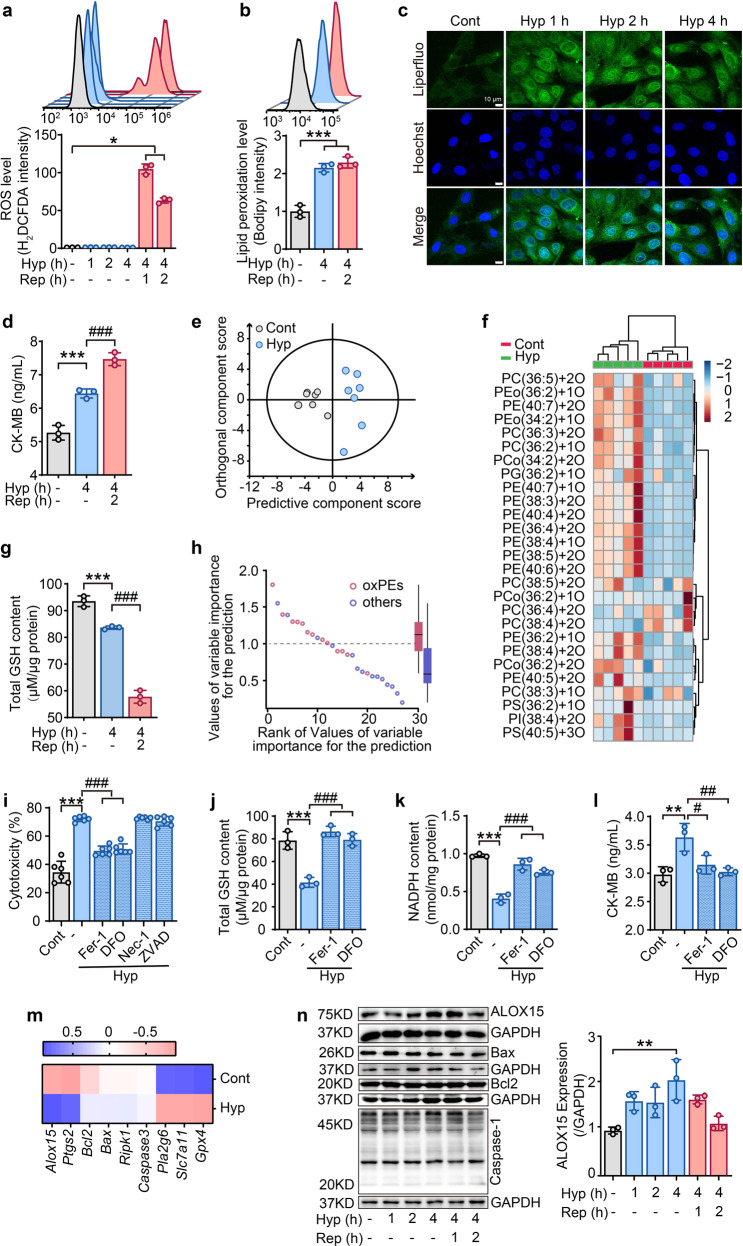
Phospholipid peroxidation is triggered by hypoxia in cardiomyocytes. **a** After hypoxia/reoxygenation, ROS levels in H9C2 cells were measured by H2DCFDA staining using flow cytometry at different time points. **b** Lipid ROS detected by flow cytometry after staining H9C2 cells with Bodipy. **c** Lipid ROS detected by confocal microscopy performed at different time periods of hypoxia in H9C2 cells stained with Liperfluo. **d** CK-MB content in H9C2 cells after hypoxia/reoxygenation. **e** Multivariate statistical analysis of oxidative phospholipids using OPLS-DA analysis between control and hypoxia-treated cells. Each point represents a sample (*n* = 7). **f** Heat map indicating changes to different oxPLs in hypoxia-exposed H9C2 cells (*n* = 5). **g** GSH content in H9C2 cells treated with hypoxia/reoxygenation. **h** Dot plots demonstrating variable importance for prediction scores of various oxPLs species in distinguishing control versus ischemia-treated H9C2 cells. PE-ox species (variable importance for prediction score>1) were the predominant oxidized species that separated the control from hypoxia treatment (*n* = 7). **i** Effect of different cell death inhibitors on the cytotoxicity of H9C2 cells exposed to 4-h hypoxia. All inhibitors were pretreated for 2 h before hypoxia treatment. Ferroptosis inhibitor: ferrostatin-1(Fer-1, 1 μM), deferoxamine (DFO, 100 μM); necroptosis inhibitor: necrostatin-1 (Nec-1, 1 μM); apoptosis inhibitor: Z-VAD-FMK (ZVAD, 1 μM). Effects of ferroptosis inhibitors on the contents of total GSH (**j**), NADPH (**k**), and CK-MB (**l**) after 4-h hypoxia. Ferrostatin-1 (Fer-1, 1 μM) and deferoxamine (DFO, 100 μM) were pretreated for 2 h before hypoxia treatment. **m** A summary heat map of quantitative RT-PCR analysis of genes related with ferroptosis (*Alox15*, *Ptgs2*, *Pla2g6*, *Slc7a11*, and *Gpx4*), apoptosis (*Bcl*_*2*_ and *Bax*), and necrosis (*Ripk1*) in H9C2 cells treated with 4-h hypoxia (*n* = 3). **n** Protein expressions for ALOX15, Bax, Bcl2 and Caspase-1 in hypoxia/reoxygenation treated H9C2 cells (*n* = 3). The right panel indicates the statistical analysis forALOX15 expression. All the quantitative data are presented as mean ± SD and statistical significance was assessed by one-way ANOVA followed by Tukey post-hoc test. *p < 0.05, **p < 0.01, ***p < 0.001 vs control (cont) group; ^#^*p* < 0.05, ^##^*p* < 0.01, ^###^*p* < 0.001 vs hypoxia (Hyp) group

Ischemia and reperfusion injury are two main phases that lead damage to the myocardium in myocardial I/R.^17^ At present, most studies focus on myocardial cell damage caused by a large amount of oxygen-free radicals during reperfusion. In order to examine the changes in redox reaction during both ischemia and reperfusion, an in vitro hypoxia/reoxygenation (H/R) model was established in cardiomyocyte H9C2 cells. Consistent with previous studies, common ROS detected by H_2_DCFDA was produced in large quantities in the reoxygenation phase but not in the hypoxia phase (Fig. 1a). By contrast, a robust level of lipid radicals was observed in the hypoxia phase, indicated by Bodipy staining (Fig. 1b). This finding was confirmed by staining with another lipid oxidation-specific probe, Liperfluo (Fig. 1c). Indeed, myocardial cell death (Supplementary Fig. 1a, b) and cell damage (Fig. 1d, Supplementary Fig. 1c, d) have already appeared in the hypoxia phase. Thus, it is inferred that ischemia has triggered a lipid redox reactions in myocardial cells, which might potentially aggravate oxidative damage in the later stage of reperfusion.

To further verify this finding, the oxidized lipids in hypoxic myocardial cells were identified using HPLC-MS-based redox lipidomics.^18,19^ Orthogonal partial least squares discriminant analysis (OPLS-DA), a multivariate analysis method for the regression modeling of multiple dependent variables to multiple independent variables^20^ identified a distinct difference of phospholipid peroxidation products (oxPLs) between hypoxic and non-hypoxic conditions of myocardial cells (Fig. 1e). Further analysis revealed that phosphatidylethanolamines (PEs) were the most predominant class of oxidized phospholipids in the hypoxic cells (Fig. 1f and h). These results suggest that oxPLs and the resultant ferroptosis are likely involved in this phase of hypoxia. Accumulation of oxPLs, especially oxidized PEs (oxPEs), is known to be critical death signals and one of the key hallmark of ferroptosis.^21^ To verify this, the samples were analyzed for the presence of two biochemical indicators of ferroptosis, glutathione (GSH) and nicotinamide adenine dinucleotide phosphate (NADPH). The results showed that these two small anti-oxidative molecules, were largely absent during the hypoxia phase (Fig. 1g and Supplementary Fig. 1e). Furthermore, two ferroptosis inhibitors ferrostatin-1 (Fer-1) and deferoxamine (DFO), an apoptosis inhibitor z-VAD-fmk (ZVAD), and a necroptosis inhibitor necrostatin-1 (Nec-1) were used to distinguish the type of hypoxia-induced cell death. As expected, ferroptosis inhibitors protected against hypoxia-provoked reduction in cell viability (Fig. 1i and Supplementary Fig. 1f), reversed the levels of GSH (Fig. 1j) and NADPH (Fig. 1k) and prevented the release of CK-MB (Fig. 1l). In comparison, apoptosis and necroptosis inhibitors presented little effect against cell death (Fig. 1i and Supplementary Fig. 1f).

Next, the expressions of several genes associated with ferroptosis, apoptosis or necrosis were examined by reverse transcription-polymerase chain reaction (RT-PCR). As shown in Fig. 1m, apoptosis and necrosis-related genes, including *Bax*, *Bcl-2*, *Ripk1* and *Caspase3*, were not affected by hypoxia. On the other hand, the mRNA levels of ferroptosis regulators, including *Alox15*, *Alox12*, *Alox5*, *Ptgs2*, *Pla2g6*, *Slc7a11* and *Gpx4*, increased or decreased (Fig. 1m). Of these changes, lipoxygenase ALOX15 expression was the most obvious, which was also confirmed by western blotting (Fig. 1n). Taken together, these data illustrate that phospholipid peroxidation is an essential feature in the hypoxia phase of myocardial cells, and the ferroptotic cell death pathway has already been initiated during hypoxia.

### Oxidized PUFA-PEs serve as crucial death signals for ischemia-induced myocardial damage

**Fig. 2:**
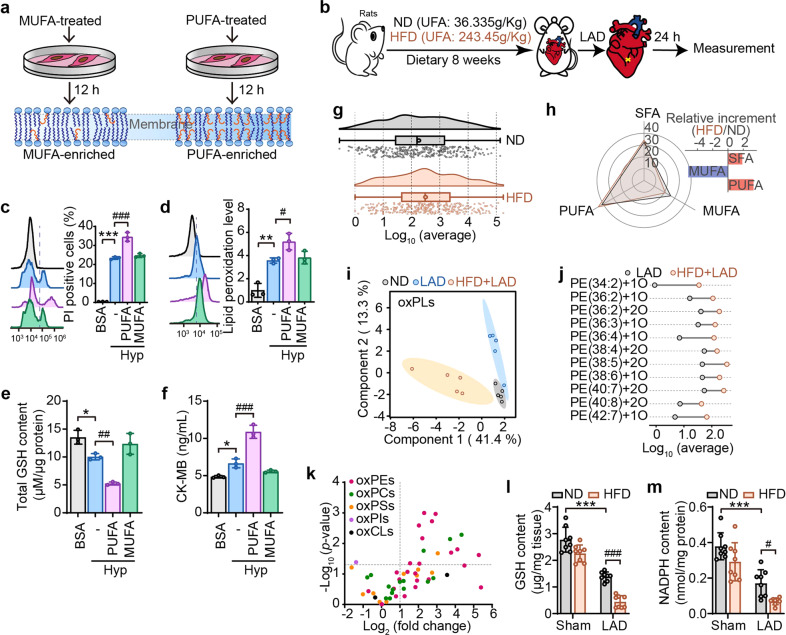
Oxidized PUFA-PEs serve as crucial death signals for ischemia-induced myocardial damage. **a** Experimental schema for establishing MUFA or PUFA-enriched cells. H9C2 cells were treated with oleic acid (OA, 80 μM) or linoleic acid (LA, 80 μM) for 12 h. MUFA, monounsaturated fatty acids; UFA, unsaturated fatty acids. **b** Schematic depicting the establishment of ischemia animal model in rats fed with normal diet (ND, containing 36.335 g/kg of UFA) or high-fat diet (HFD, containing 243.45 g/kg of UFA). After 8 weeks-feeding, rats were subjected to ischemia by left anterior descending (LAD) coronary artery ligation. After inducing hypoxia in cells treated with BSA-prepared PUFA or MUFA, cell death (**c**) and lipid peroxidation (**d**) were, respectively, evaluated with PI and Liperfluo staining followed by flow cytometry. BSA treatment was set as the control group. Total content of GSH (**e**) or CK-MB (**f**) in PUFA/MUFA-enriched cells at 4-h hypoxia treatment. **g** The raincloud plot shows the content of Log10 (average) changes in total PUFAs in the heart of ND-fed and HFD-fed rats (*n* = 5). **h** Radar chart indicates the changes of SFA, MUFA, and PUFA in the heart of ND-fed and HFD-fed rats. The relative increment of these fatty acids was represented by a bar chart (*n* = 5). **i** OxPLs were extracted and interpreted by principal component analysis (PCA), and the 2D score plots display repertoires of ND-sham, ND-LAD, and HFD-LAD rats. Each point represents a sample, and ellipses represent 95% confidence regions (*n* = 5). **j** Dumbbell chart shows different types of oxPEs in the content of Log10 (average) changes among LAD-ligated rats fed with ND or HFD (*n* = 5). **k** Volcano plots showing changes in oxPLs in heart tissue of LAD ligated rats with ND-and HFD-fed (*n* = 5). The significance of -log10 (*P* value) by *t* test. Content of GSH (**l**) and NADPH (**m**) were detected in ND or HFD-fed rats after 24-h LAD ligation. All the quantitative data are presented as mean ± SD and statistical significance was assessed by one-way ANOVA followed by Tukey post-hoc test. For the in vitro experiments, *p < 0.05, **p < 0.01, ***p < 0.001 vs BSA control group; ^#^*p* < 0.05, ^##^*p* < 0.01, ^###^*p* < 0.001 vs hypoxia (Hyp) group. For the in vivo experiments, ***p < 0.001 vs “ND + sham” group, ^#^*p* < 0.05, ^###^*p* < 0.001 vs “ND + LAD” group

Since the increase of PUFA at the bis-allylic position of phospholipid is a driving factor for peroxidation and ferroptosis,^22^ a PUFA-enriched cell model was established by incubating H9C2 cells with linoleic acid (LA, Fig. 2a). Cells enriched with oleic acid (OA) were used as monounsaturated fatty acids (MUFA) controls (Fig. 2a). The impact of fatty acids on cell viability was determined, based on which the treatment conditions for LA and OA (80 μM, 12 h) was chosen (Supplementary Fig. 2a, b). Oil red O staining showed that LA and OA were successfully incorporated into cells (Supplementary Fig. 2c). As expected, LA challenge enhanced the sensitivity of myocardial cells to hypoxia-induced death (Fig. 2c), lipid peroxidation (Fig. 2d), GSH depletion (Fig. 2e), and CK-MB elevation (Fig. 2f). In sharp contrast to PUFA-enriched cells, MUFA enriched cells exerted little impact on these parameters. These results infer that the increase of PUFAs indeed aggravates the hypoxia-induced injury of myocardial cells.

To determine whether increase in PUFA is relevant in vivo, PUFA-enriched animal models were established in left anterior descending (LAD)-treated Wistar rats by feeding a PUFA-containing high-fat-diet (PUFA-HFD) for 8 weeks (Fig. 2b, Supplementary Fig. 2d–m). Results showed that PUFA-HFD exposure induced a shift of total PUFAs, evidenced by raincloud plot analysis (Fig. 2g). Radar chart indicated that PUFA-HFD feed resulted in a rise of about 3% in PUFAs and a fall of about 6% in MUFA (Fig. 2h). Subsequently, LC-MS/MS-based redox lipidomics analysis was performed in heart tissues by subjecting them to LAD ligation in normal diet (ND) and HFD-fed rats, with internal standard substances as quality control (Supplementary Fig. 3 and Supplementary Fig. 4). A total 410 individual phospholipids species, including 339 non-oxygenated and 71 oxygenated phospholipids were detected. Furthermore, principal component analysis (PCA), a mathematical procedure that decreases the dimensionality of data while preserving the majority of the variance in the dataset by transforming it into principle components using an orthogonal transformation,^23^ indicated that oxPLs variables were distinguishable between ND and HFD groups (Fig. 2i). Oxidized phospholipids were qualitatively and semi-quantitively analyzed and five major classes of oxygenated phospholipids, including PEs, phosphatidylcholines (PCs), phosphatidylserines (PSs), phosphatidylglycerols (PGs), phosphatidylinositols (PIs) and cardiolipins (CLs) were identified (Fig. 2k). Intriguingly, among these oxidized phospholipids, oxPEs, which has been implicated as a ferroptosis-specific cell death signal,^22,24^ were the most significantly altered and were identified to be the primary contributor to the total difference (Fig. 2j, k, Supplementary Fig. 5a).

Unexpectedly, PUFA-HFD feed rats showed significant susceptibility to myocardial ischemia, exhibited by increased higher mortality (Supplementary Fig. 5b), deteriorated cardiac function (Supplementary Fig. 5c–i and 5k-l), and aggravated pathological damage (Supplementary Fig. 5j). PUFA-HFD feed also had significant impact on lipid peroxidation-related parameters, including diminished contents of GSH (Fig. 2l) and NADPH (Fig. 2m), and elevated malondialdehyde (MDA) level (Supplementary Fig. 5m) in heart tissues after LAD ligation. These data highlight the relevance of oxidized PEs-triggered ferroptosis and ischemia-induced myocardial damage.

### ALOX15 expression is highly correlated with ischemia/hypoxia-induced cardiomyocyte damage

**Fig. 3:**
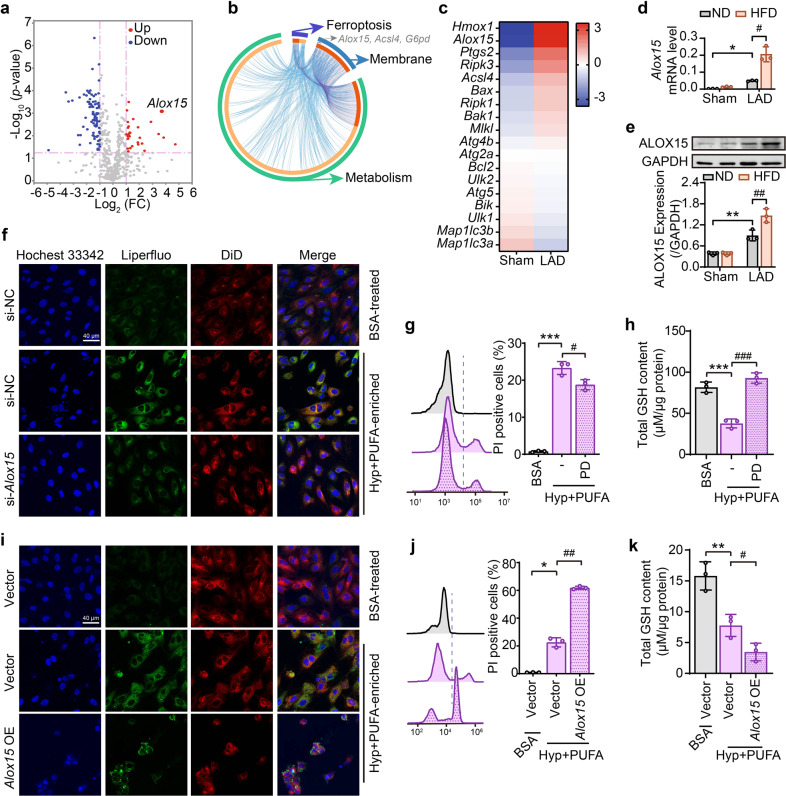
ALOX15 expression is highly correlated with ischemia/hypoxia-induced cardiomyocyte damage. **a**–**c** The differential genes analysis in the heart tissues of sham and ischemia hearts. **a** The volcano plots express the differentially expressed genes between the two groups in metabolic pathways by KEGG analysis. The genes upregulated (red point) or downregulated (blue point) at least the absolute value of log2 (fold change) >1 and *P* < 0.05 was set as the thresholds between any two treatments (*n* = 4). **b** The Circos plot indicates overlapped differential genes across ferroptosis, membrane and metabolic pathways. Outside of the Circos, each arc represents an identity pathway gene list, and inside, a spot on the arc represents a gene. The greater the number of purple links and the longer the dark orange arcs imply a more significant overlap among the input gene lists. *Alox15*, *Acls4* and *G6pd* were identified as the overlapping genes of three pathways. **c** Heat map of differential expressions of classical genes related to ferroptosis (*Hmox-1*, *Alox15*, *Ptgs2*, *Acsl4*), apoptosis (*Bax, Bcl*_*2*_, *Bik*, *Bak1*), necrosis (*Ripk3*, *Mlkl*, *Ripk1*), and autophagy (*Atg4b*, *Atg2a*, *Ulk2*, *Atg5*, *Ulk1*, *Map1lc3b*, *Map1lc3a*). Color scales show the differences in expression of each gene in the indicated sample relative to its expression in log_2_ (fold change). The mRNA (**d**) and protein (**e**) levels of ALOX15 in heart tissues of ND-or HFD-fed rats after 24-h LAD ligation and the semi-quantification is shown in the lower panel. **f** After si-*Alox15*, lipid peroxidation was detected by confocal microscopy after Liperfluo staining in PUFA-enriched H9C2 cells under hypoxia. Effect of ALOX15 inhibitor PD 146176 (PD, 10 μM) on cell death (**g**) and GSH content (**h**) was assessed by PI staining in PUFA-enriched H9C2 cells under hypoxia. After *Alox15* overexpression (*Alox15* OE) in PUFA-enriched H9C2 cells under hypoxia, lipid peroxidation (**i**), cell death (**j**) and GSH content (**k**) were, respectively, determined by confocal microscopy, flow cytometry and HPLC-MS. All the quantitative data are presented as mean ± SD and statistical significance was assessed by one-way ANOVA followed by Tukey post-hoc test. For the in vitro experiments, *p < 0.05, **p < 0.01, ***p < 0.001 vs control (Cont) group; ^#^*p* < 0.05, ^##^*p* < 0.01, ^###^*p* < 0.001 vs hypoxia (Hyp) group. For the in vivo experiments, *p < 0.05, **p < 0.01 vs “ND + sham” group, ^#^*p* < 0.05, ^##^*p* < 0.01 vs “ND + LAD” group

To identify the molecular players of regulating phospholipid peroxidation during myocardial ischemia, RNA-seq analysis was performed in heart tissues from sham and LAD-ligation ischemic rats. Gene enrichment analysis for differential gene expressions revealed that metabolism-related pathways were largely impacted (Supplementary Fig. 6a). Apparent changes of genes were also noted in pathways related with membrane, integral component of plasma membrane, oxidation-reduction process, or response to hypoxia (Supplementary Fig. 6b). Notably, some differential genes overlapped across ferroptosis, membrane, and metabolic pathways (Fig. 3b). Next, leading-edge genes enriched in metabolism-related pathways between sham and ischemia hearts were examined to reveal that ALOX15, a core enzyme in phospholipid peroxidation and ferroptosis was greatly upregulated at the gene expression and protein levels under ischemia conditions (Fig. 3a, c). It is worth noting that the expression of several marker genes involved in apoptosis, necrosis and autophagy were not affected by ischemia (Fig. 3c and Supplementary Fig. 6c). As expected, LAD ligation-induced ischemia increased gene and protein levels of ALOX15, and HFD augmented this change (Fig. 3d, e).

To further establish a direct correlation between ALOX15-mediated phospholipid peroxidation and myocardial ischemia, ALOX15 inhibitors (PD 146176 and baicalein) and, gain-of-function and loss-of-function approaches were employed in H9C2 cells. In normal cells or PUFA-enriched cells, ALOX15 inhibition provided protection against cell death (Fig. 3g), while ALOX15 overexpression exacerbated the population of PI-stained cells (Fig. 3j). On the other hand, ALOX15 inhibition or knockdown (Supplementary Fig. 6d) was able to not only limit cell death and lipid peroxidation (Fig. 3f, Supplementary Fig. 6e, f), but also increase GSH content (Fig. 3h) in the PUFA-enriched cells. Conversely, *Alox15* overexpression by plasmid transfection (Supplementary Fig. 6d) showed an opposite regulation in lipid peroxidation (Fig. 3i and Supplementary Fig. 6g) and GSH level (Fig. 3k). These results illustrate a significant role for ALOX15 in ischemia/hypoxia-provoked phospholipid peroxidation and the resultant cardiomyocyte damage.

### *Alox15*^–/–^ mice lose PUFA-induced susceptibility towards ischemia-induced myocardial damage

**Fig. 4:**
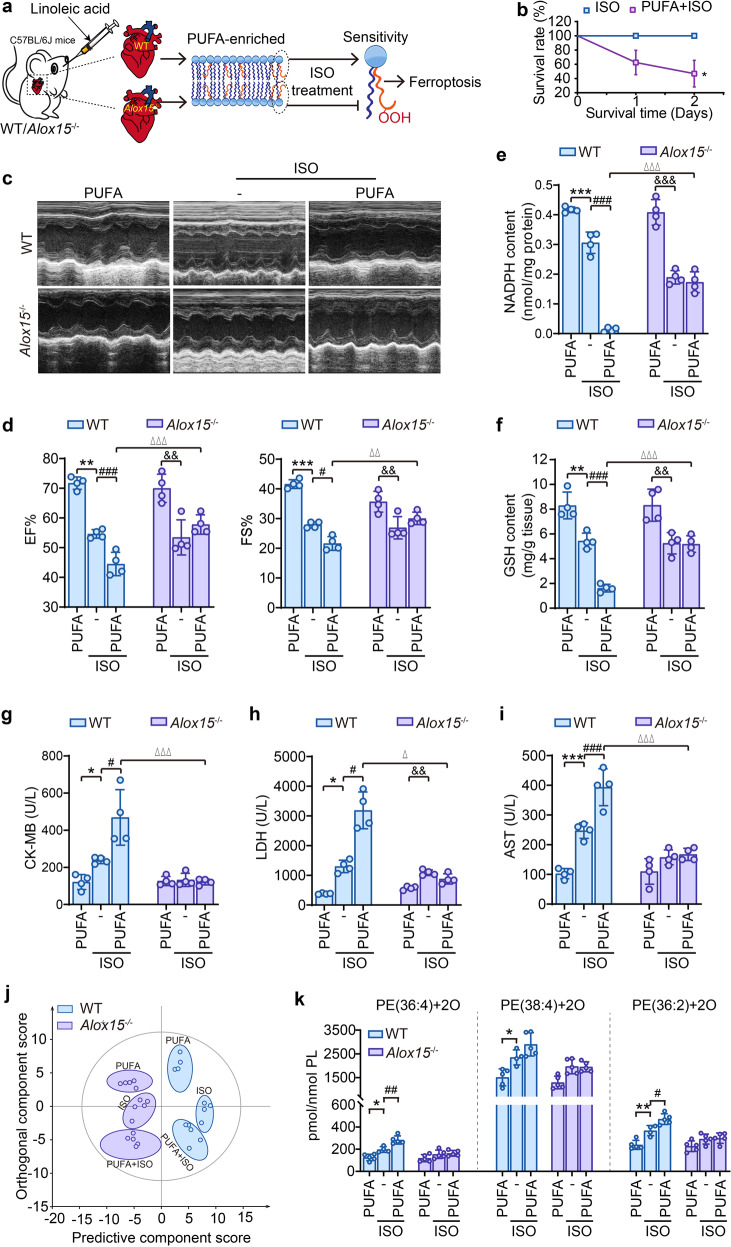
*Alox15*^–/–^ mice lose PUFA-induced susceptibility towards ischemia-induced myocardial damage. **a** Experimental schema for establishing ischemia animal model in PUFA-enriched in WT or *Alox15*^–/–^ mice. All animals were intragastrically administered with LA (2 g/kg, 14 d) and then intraperitoneally injected isoproterenol (ISO), 40 mg/kg. The heart tissues were obtained one day after ISO injection. **b** Influence of PUFA on the survival rate of WT mice with ISO treatment (*i.p*., 40 mg/kg). *p < 0.05 by Log-rank (Mantel-Cox) test (*n* = 8). **c** and **d** Effect of PUFA on heart function of WT or *Alox15*^–/–^ mice with ISO treatment. **c** Representative echocardiography images. **d** The left ventricular ejection fraction (EF%) and left ventricular shortening fraction (FS%). The contents of NADPH (**e**) and GSH (**f**) were detected in heart tissues of ISO-injected WT or *Alox15*^–/–^ mice. The levels of myocardial enzymes, including CK-MB (**g**), LDH (**h**) and AST (**i**) were analyzed in heart tissues of ISO-injected WT or *Alox15*^–/–^ mice. **j** OPLS-DA score plots analyze the separation of oxidized oxPLs between WT and *Alox15*^–/–^ mice. Each point represents a sample (*n* = 4–5). **k** Quantitative data for di-oxygenated PE species, including PE (36:4), PE (38:4) and PE (36:2) in heart tissues of ISO-injected WT or *Alox15*^–/–^ mice. All the quantitative data are presented as mean ± SD and statistical significance was assessed by one-way ANOVA followed by Tukey post-hoc test (**d**–**i**) or *t* test (**k**). *p < 0.05, **p < 0.01, ***p < 0.001 vs the “WT + PUFA” group; ^#^*p* < 0.05, ^##^*p* < 0.01, ^###^*p* < 0.001 vs “WT + ISO” group. ^&^*p* < 0.05, ^&&^*p* < 0.01, ^&&&^*p* < 0.001 vs “*Alox15*^–/–^+PUFA” group; ^△^*p* < 0.05, ^△△^*p* < 0.01, ^△△△^*p* < 0.001 vs “WT + PUFA + ISO” group

To verify the essential role of ALOX15 in ischemia-induced myocardial damage, an ischemia model was established by isoproterenol (ISO) treatment in PUFA-enriched *Alox15*^–/–^ and WT mice (Fig. 4a). For the preparation of PUFA-enriched animals, LA (2 g/kg) was administered via intragastric route to mice for 2 weeks. Following LA challenge, the animals were intraperitoneally injected with 40 mg/kg ISO to induce ischemia. PUFA-enriched mice showed an increased susceptibility towards ischemia-induced myocardial damage, represented by lowered survival (Fig. 4b), exasperated cardiac function (Fig. 4c, d) as well as raised serum levels of creatine kinase isoenzyme-MB (CK-MB), lactate dehydrogenase (LDH) and aspartate aminotransferase (AST) (Fig. 4g–i). In line with hypoxia-exposed cardiocytes, we further provided in vivo proof demonstrating the impossibility of ischemia in inducing apoptosis and necrosis in ISO-established animal model (Supplementary Fig. 7). Noticeably, PUFA-augmented myocardial damage in ISO mice were abolished in *Alox15*^–/–^ mice. Echocardiographic images presented a recovered cardiac function in *Alox15* knockout mice, with a significant reduction in EF% and FS% compared with PUFA control mice (Fig. 4c, d). In parallel, PUFA-promoted release of myocardial function-related enzymes in blood were also rescued by ALOX15 deficiency (Fig. 4g–i). The insensitivity of *Alox15*^–/–^ mice to ischemia was related to phospholipid peroxidation as inferred by the observation that in rat ischemia induced by LAD. PUFA treatment also depleted NADPH and GSH in the heart tissues for ISO-injected mice, which was abrogated by *Alox15* knockout (Fig. 4e, f). Moreover, an OPLS-DA model constructed using oxPLs profiles to discriminate different groups (Fig. 4j) revealed that *Alox15*^–/–^ mice and WT mice could be distinguished based not only on oxPLs but also different treatments within the same genotype of mice. This indicated that oxPLs induced by ISO treatment are susceptible to high-fat diet and perturbation of ALOX15. Among the 45 oxidized PEs detected, PE (36:4)+2 O, PE (38:4)+2 O and PE (36:2)+2 O, were markedly enhanced in PUFA-enriched hearts of WT mice (Fig. 4k). In contrast, *Alox15* knockout abolished PUFA-conferred increase in oxidized PEs (Fig. 4k). Taken together, these data provide suggest the in vivo relevance of a pivotal role for ALOX15-mediated phospholipid peroxidation in PUFA-mediated susceptibility to myocardial ischemia.

### Natural ALOX15 inhibitor daidzein prohibits ischemia-induced myocardial damage

**Fig. 5:**
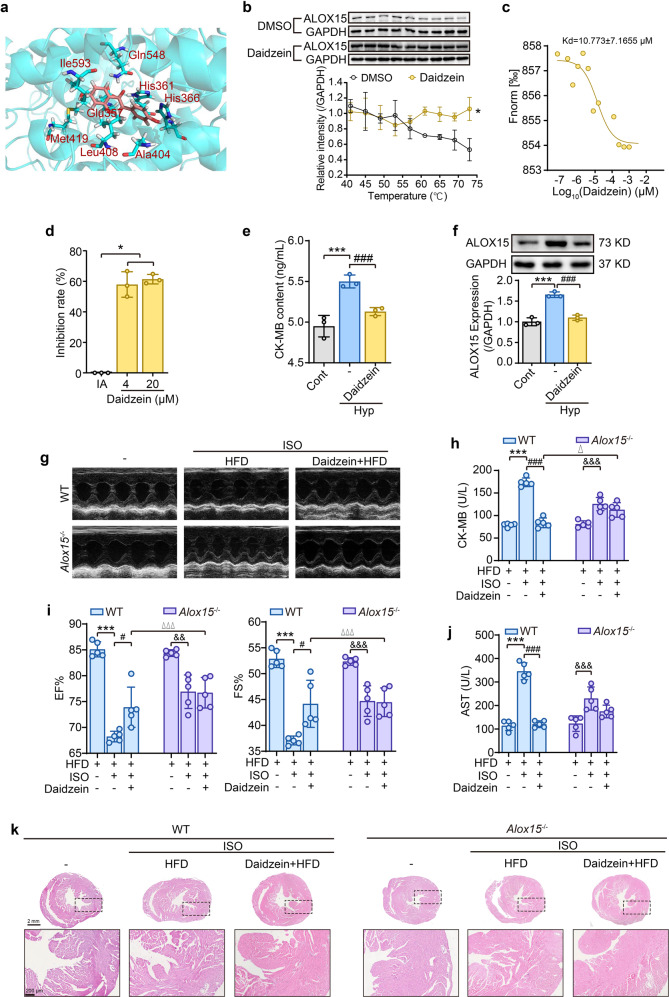
Natural ALOX15 inhibitor daidzein prohibits ischemia-induced myocardial damage. **a** Representative docking images of ALOX15 and daidzein. **b** and **c** The binding affinity of daidzein and ALOX15 was determined by CETSA and MST experiments. CETSA was performed in the lysates of ALOX15-overexpressing HEK293T cells (**b**). Data is expressed as mean ± SD, and statistical significance was analyzed by two-way ANOVA followed by the Tukey post-hoc test. *p < 0.05 vs the DMSO group. Data points in MST analysis indicate the difference in normalized fluorescence (‰) generated by daidzein binding to ALOX15, and curves show the calculated fits (**c**). **d** The inhibition rate of daidzein on ALOX15 enzyme activity. Data is expressed as mean ± SD, and statistical significance was analyzed by one-way ANOVA followed by the Tukey post-hoc test. *p < 0.05 vs Initial Activity (AI) group. Effect of daidzein on CK-MB content (**e**) and protein expression (**f**) in hypoxia H9C2 cells. Daidzein (50 μM) was pretreated for 12 h. Data is expressed as mean ± SD, and statistical significance was analyzed by one-way ANOVA followed by the Tukey post-hoc test. ***p < 0.001 vs control (Cont) group; ^###^*p* < 0.001 vs hypoxia (Hyp) group. **g–k** Effect of daidzein on heart functions and histological changes of HFD-fed WT or *Alox15*^–/–^ mice subjected with ISO. **g** Echocardiography images. **h** Serum CK-MB content. **i** The left ventricular ejection fraction (EF%) and left ventricular shortening fraction (FS%). **j** serum AST level. **k** H&E pathological examination of heart sections (*n* = 3). Data is expressed as mean ± SD, and statistical significance was analyzed by one-way ANOVA followed by the Tukey post-hoc test. ***p < 0.001 vs “WT + HFD” group, ^#^*p* < 0.05, ^###^*p* < 0.001 vs “WT + ISO” group; ^&&^*p* < 0.01, ^&&&^*p* < 0.001 vs “*Alox15*^–/–^+HFD” group; ^△^*p* < 0.05, ^△△△^*p* < 0.001 vs “WT + HFD + ISO” group

Given the importance of lipoxygenase ALOX15 in ischemia injury, a molecular docking analysis with ALOX15 was performed using more than 40,000 natural products. Using this approach, daidzein was identified with an outstanding binding affinity. Daidzein forms intermolecular hydrogen bonds with amino acid residues of His361and Gln548 and has hydrophobic interactions with Glu357, His361, His366, Ala404, Leu408, Met419, and Ile593 in ALOX15 (Fig. 5a). Interestingly, His361 had both intermolecular hydrogen bonds and hydrophobic effects (Fig. 5a). Subsequently, we performed a Cellular Thermal Shift Assay (CETSA) and found that the daidzein attenuated the degradation rate of ALOX15 with increase of temperature (Fig. 5b). The binding affinity of daidzein and lipoxygenase was further confirmed using the microscale thermophoresis assay (MST) (Fig. 5c) which revealed that daidzein dramatically inhibited ALOX15 activity (Fig. 5d). Thus, these data singled out daidzein as a potent ALOX15 inhibitor.

The protective effect of daidzein was firstly examined in vitro and results showed that it significantly reduced CK-MB level (Fig. 5e) and suppressed the expression of ALOX15 (Fig. 5f) in myocardial cells exposed to hypoxia. Additionally, daidzein displayed remarkable properties of an antioxidant (Supplementary Fig. 8). To further verify the effect of daidzein in vivo, 40 mg/kg of daidzein was intragastrically administered for 4 weeks to HFD-fed *Alox15*^–/–^ or HFD-fed WT mice. The treatment of daidzein was found to markedly reverse ischemia injury induced by PUFA-HFD and ISO treatment, as indicated by improved cardiac function (Fig. 5g, i), reduced myocardial enzymes mice (Fig. 5h, j) and attenuated pathological changes (Fig. 5k) in heart tissues of WT mice. The protective effect of daidzein on myocardial damage was abolished in *Alox15*^–/–^ mice (Fig. 5g–k). Taken together, it is concluded that the natural ALOX15 inhibitor daidzein holds potential for treating ischemia injury, and targeting ALOX15 provides an early intervention strategy for therapy of I/R.

## Discussion

Since the discovery of the lethal effects of reperfusion injury, there has been a passionate interest devoted to developing strategies to prevent reperfusion injury, and the importance of the ischemic phase to this process has been largely ignored. Although the area of myocardial damage significantly decreases after reperfusion reconstruction, it still leads to irreversible damage to at least 25% of the myocardial cells.^17^ It is now being increasingly recognized that the degree of ischemia damage could predict and determine the following reperfusion injury.^7,17,25^ Indeed, cardioprotective interventions applied during ischemic episodes, including therapeutic hypothermia^9^ and remote ischemic perconditioning,^10^ are efficient to reduce morbidity and mortality in I/R. Nonetheless, the intrinsic molecular determinants of ischemia injury remain largely unknown. In the present study, transcriptomic analysis combined with serial functional validations revealed that a key enzyme in oxidizing PUFA-phospholipids, ALOX15, was induced by ischemia/hypoxia. We also demonstrated that the contribution of ALOX15 to ischemia injury was dependent on its well established peroxidizing activity that results in accumulation of oxPEs with subsequent occurrence of ferroptosis. This surprising discovery shifts attention from common free radicals to lipoxygenase-mediated specific-oxidative response, thus overturning the long-held opinion that oxidative damage is only presented at the reperfusion stage.

Inflammation has long been recognized as one of the critical mechanisms in myocardial ischemia injury^26^ and ALOX15 has a significant impact on inflammation via producing lipid mediators.^27^ In view of no disturbed inflammatory-related pathway in RNAseq analysis and little inflammatory infiltration in H&E histological examination, we exclude the possibility of inflammation in the early stage of ischemia, which is supported by previous studies showing a significant inflammatory response only elicited until 3 days after LAD ligation.^28,29^ This finding raises the possibility that ALOX15-mediated peroxidation of PUFA-phospholipids, which is earlier than the formation of inflammatory precursors, is a crucial initial element in the pathological mechanism of ischemia injury.

For decades, the intake of PUFAs has been believed to be beneficial for heart function.^30–32^ Recently, researchers have gradually realized that the advantage of PUFAs in preventing myocardial damage might be overstated in some circumstances.^33–35^ Particularly, a large-scale randomized controlled trial investigating the effects of marine omega-3, a type of PUFAs on cardiovascular outcomes revealed an increased risk of atrial fibrillation.^36^ These unexpected clinical evidences raise the necessity of unearthing the more adverse aspects of PUFAs in cardiovascular diseases. More intriguingly, the increasing PUFAs in plasma of obese patients have been reported to correlate well with myocardial damage.^37^ In line with this clinical observation and an earlier report in animals,^38^ our findings found that a reconstituted composition of fatty acids in the cardiomyocytes of heart tissues of HFD-fed mice, led to increase in PUFAs and a decrease in MUFAs. We further determined that PUFA-enriched animals/cells were prone to ischemia/hypoxia injury due to the induction of ALOX15. Our study, for the first time, provides a causal link between PUFAs intake and myocardial ischemia damage mediated by ALOX15-induced phospholipid peroxidation.

The involvement of various forms of cardiomyocyte cell death, such as apoptosis, necroptosis and pyroptosis in I/R injury has been traditionally recognized.^39,40^ For instances, autophagy was found to be induced at 8 days post LAD-ligation,^41^ while apoptosis occurred at 4 weeks post LAD-ligation.^42^ Necrosis tends to occur in the reperfusion phase of I/R.^43^ Nevertheless, in our study, short-term ischemia was proved to exert little impact on apoptosis and necrosis in both ISO and LAD ischemia models. In contrast to these classic types of cell death, we unearth the essential role of ferroptosis in the ischemia phase of I/R. Due to the central role of oxidative response in I/R injury, the association between ferroptosis and heart function has undoubtedly attracted much attention. It has been recently reported that ferritin H/SLC7A11 promotes ferroptosis in cardiomyopathy,^44^ and iron chelators prevented myocardial ischemia-reperfusion injur.^15,16^ While these studies have raised concerns on how ferroptosis affect the pathophysiology of I/R at the molecular level, they mainly focused on perturbances in the reperfusion phase. The data presented here focused on the ischemia phase of I/R demonstrating unequivocally that induction of ALOX15 triggers oxidization of PUFA-phospholipids, especially PUFA-PEs leading to cardiomyocyte ferroptosis. We therefore believe this to be a priming signal that augments robust oxidative damage in the reperfusion phase.

Although there have been many attempts to develop new therapies against I/R, these effects have been thwarted by limitations in the availability of effective drugs. In this respect, our finding on the role of ALOX15 as a potential therapeutic target of ischemia injury led to identification of daidzein as a potent ALOX15 inhibitor, which shows promise in the treatment for both phases of I/R injury. Recently, increasing investigations have been devoted to developing new clinical biomarkers of myocardial damage^45–47^ and promoting early diagnosis of I/R.^48–50^ It is predictive that the discovery of key regulators in ischemia stage, such as ALOX15 we identified in the present study, may also contribute to exploiting biomarkers for the early diagnosis of I/R.

A pending question remains to be answered is that how ALOX15 in ischemic heart tissues. In responses to ischemia, cellular synthesis of proteins, especially those related with oxidation-reduction reaction, are activated to coordinate diverse biological outputs and fine-tune the responses to hypoxic stress. Prior to our study, there have been clinical observations indicating a high expression of ALOX15 in both acute and long-term ischemic hearts of patients.^51,52^ Meanwhile, ALOX15 was also reported to abundantly expressed in macrophages under hypoxia condition.^53,54^ What’s more, parallel enhanced expressions of hypoxia-inducible factor 1 (HIF-1) and ALOX15 in clinical ischemic heart samples implied that ALOX15 transcription might be associated with HIF-1.^55^ Nevertheless, this conclusion requires more direct and convincing evidences in future investigations.

In summary, the present study utilized PUFAs-enriched in vivo and in vitro models to recapitulate phospholipid peroxidation and ferroptosis as essential features of ischemia-induced myocardial damage. The induction of ALOX15 was identified as a determinant of susceptible factor in PUFAs-induced ischemia. These findings including the identification of daidzein as an inhibitor open up new horizons in the understanding of pathogenesis in ischemia damage, paving the way for development of early therapeutic strategies in I/R.

## Materials and methods

### Cell culture and establishment of in vitro ischemia model

Rat cardiac myoblast cell line H9C2 cells (American Type Culture Collection, Manassas, Virginia, USA) were cultured in high-glucose DMEM supplemented with 10% (v/v) FBS, penicillin G (100 U/ml), and streptomycin (100 μg/ml), under 5% CO_2_ at 37 °C.

To establish an in vitro ischemia model, H9C2 cells were placed in the Ischemia Modular Incubator Chamber (Billups-Rothenberg Inc, USA), filled with mixed gas (5% CO_2_ and 95% N2). Cells in this chamber were cultured at 37 °C for 4 h.

### Preparation of PUFA-enriched cells

PUFA-enriched cells were prepared as described previously.^56^ Briefly, H9C2 cells were plated into dishes and cultured for 24 h in complete medium (DMEM, 10% FBS) containing BSA-conjugated 80 μM LA.

### Animals and treatment

Male Wistar rats (Laboratory Animal Center, Southern Medical University, Guangzhou, China) weighing 120–130 g were maintained in a temperature-controlled room with light-dark cycles of 12 h:12 h. They were given free access to either a HFD (40% carbohydrate, 20% protein and 40% fat by calories) or a ND (70% carbohydrate, 20% protein and 10% fat by calories) for eight weeks.

*Alox15*^–/–^ mice were purchased from Jackson Laboratory (Stock No. 002778) and wild-type C57BL/6 J mice were obtained from Laboratory Animal Center, Southern Medical University. These animals were maintained ingroup and housed under pathogen-free conditions. Each mouse received standard chow of 3 g/day and unlimited drinking water. The Laboratory Animal Committee of Jinan University has approved all animal experiments (No. 2019358).

### Establishment of myocardial ischemia model in rats

LAD coronary artery-ligation surgery was employed to establish myocardial ischemia model according to previous studies.^57^ In brief, Wistar rats were anesthetized with 5% isoflurane in the animal anesthetic machine (Shanghai Yuyan Instruments Co., Ltd., Shanghai, China), and 2% isoflurane was maintained throughout the operation. A 14-gauge angiocatheter was applied for endotracheal intubation and connected to mechanical ventilation (RWD Life Science Co., Ltd, Shenzhen, China). The animal electrocardiogram (ECG) system (PowerLab Multiconductor Physiograph, ADInstruments Ltd., Australia) was used to monitor heart function of rats. The thoracotomy was performed at the left fourth intercostal space, and the heart was permanently ligated around the left anterior descending artery 2 mm below the left atrium with 6-0 Polypropylene sutures. Ischemia was verified by an elevated ST segment in ECG and color change in the myocardial tissue of the ischemic area. The thoracotomy incision was closed in layers and the endotracheal tube was removed. Buprenorphine (0.5 mg/kg) was used for postoperative pain control.

### Measurement of myocardial infarct size

The hearts of rats were harvested and perfused in phosphate buffer saline after a 24-h LAD-ligation period. These tissues were frozen at –20 °C for 30 min, cut transversely into four sections of 2 mm thickness each, incubated with 2% triphenyl tetrazolium chloride (TTC; Aladdin, Shanghai, China) in phosphate solution for 10 min at 37 °C, and then fixed by 10% paraformaldehyde for 24 h. The unstained tissue reveals the infarcted area, while TTC-stained tissue in red color represents the non-infarcted region. These sections were digitally photographed and the infarct size was analyzed using ImageJ and expressed as the percentage of infarcted area relative to non-infarcted area as described previously.^58,59^

### Establishment of myocardial ischemia model in LA-treated *Alox15*^–/–^ and WT mice

Each *Alox15*^–/–^ and WT C75BL/6 J mouse was intragastrically administered with 50 mg LA every day. The amount of LA was according to daily intake of equal calories of 110 g fat for 60 kg human.^60^ Two weeks later, mice were received 40 mg/kg ISO by intraperitoneally injection to induce myocardial ischemia.

### Echocardiography

Echocardiography was utilized to detect the heart function of ischemic rats or mice. During the test, anesthesia of animals were maintained by inhalation of 2% isoflurane. The echocardiography was performed using a Visual Sonics Vevo 2100 (VisualSonics, Canada) taken from M‐mode. The heart function was obtained from long and short axes at the level of the papillary muscles in the parasternal. In addition, left ventricular (LV) end-diastolic dimension, LV end-systolic dimension (LVDs), interventricular septum thickness at diastole (IVSd), and LV posterior wall thickness at diastole (LVPWd) were calculated for ejection fraction (EF%), fractional shortening (FS%), and left ventricular mass.

### Histology and immunohistochemistry

The hearts harvested from ischemic mice or rats were fixed in 4% paraformaldehyde overnight, buffered with 1x PBS, dehydrated in stepwise ethanol, and transferred to xylene before being embedded in paraffin. The paraffin-embedded tissues were sliced into 4 µm sections, and the histological changes were examined by Masson staining (Leagene Biotech Co., Ltd., Beijing, China), and H&E staining (Beyotime Biotech Co., Ltd., Shanghai, China). These histological changes were visualized and analyzed by Scanning Microscope (PreciPoint, Germany).

### The detection of ROS and lipid peroxidation in cells

Harvested at the density of 10 × 10^6^, H9C2 cells were stained with H2DCFDA (MedChemExpress, China) to detect ROS level, and stained with Bodipy (BODIPY™ 581/591 C11, Invitrogen, USA) or Liperfluo (Dojindo, Shanghai, China) to detect lipid peroxidation level. The signals were collected using by flow cytometry (CytoFLEX S equipped with Kaluza analysis 2.1, Beckman coulter, USA) or imaged by an Olympus FV3000 confocal laser scanning microscope (Olympus, Japan).

### Preparation of phospholipids and LC-MS/MS-based phospholipidomics analysis

The preparation and analysis of phospholipids were as described previously.^18^ Phospholipids were analyzed by LC-MS using a Dionex Ultimate 3000 HPLC system coupled with a Q-Exactive Hybrid Quadrupole-Orbitrap mass spectrometer (Thermo Fisher Scientific) using a normal phase column (Luna 3 μm) Silica (2) 100 Å, 150 × 2.0 mm, (Phenomenex). The column temperature was set at 35 °C. The analysis was carried out using gradient solvents (A and B) containing 10 mM ammonium formate at a flow rate of 0.2 mL/min. Solvent A contained isopropanol/hexane/water (285: 215: 5, V/V/V), and solvent B contained isopropanol/hexane/water (285:215:40, V/V/V). All solvents were LC/MS-grade. The gradient elution program was set as follows: 0 min, 10% B; 23 min, 32%; 32 min, 65%; 35 min, 100%; 70 min, 100%. Analysis was performed in negative ion mode at a resolution of 70,000 for the full MS scan and 17,500 for the MS^2^ scan in data-dependent mode. The scan range for MS analysis was m/z 400–1800 with a maximum injection time of 200 ms using 1 microscan. Capillary spray voltage was set at 3.0 kV, and capillary temperature was 320 °C. The S-lens Rf level was set to 60. A maximum injection time of 500 ms was used for MS^2^ (high-energy collisional dissociation) analysis with collision energy set to 24. An isolation window of 1.0 Da was set for the MS and MS^2^ scans.

Peaks with a signal-to-noise ratio of more than three were identified and searched against in-house an oxidized phospholipid database. Values for m/z were matched within 5 ppm to identify the lipid species. Lipids were further filtered by retention time and confirmed by MS^2^ analysis with the fragments used for their identification (https://www.lipidmaps.org/). Quantitative analysis is based on calibration curves generated by known amounts of reference standards and its corresponding internal standards, including CL(16:0/18:2/18:2/20:4), PC(18:1/18:1), PE(18:1/18:1), PG(18:1/18:1), PI(18:1/18:1), PS(18:1/18:1), CL(14:0/14:0/14:0/14:0), PC(16:0-d31/18:1), PE(16:0-d31/18:1), PG(16:0-d31/18:1), PI(16:0-d31/18:1), PS(16:0-d31/18:1). The sn-position of acyl chains in PL was assigned based on the intensities of carboxylate anions and the fragments of loss of sn-2 acyl chain as ketene (R_2_CH=C=O) in MS/MS spectra.^61,62^

### Flow cytometric analysis of PI staining

Cells were grown on dishes at the density of 10 × 10^6^. After ischemia establishment, 0.05 mg/mL of propidium iodide (PI) (Sigma) was added to stain dead cells and analyzed by flow cytometry (CytoFLEX S equipped with Kaluza analysis 2.1, Beckman coulter, USA).

### Genome-wide RNA sequencing and analysis

Total RNA from infarct tissue was isolated from control and LAD surgery rats after a 24-h myocardial injury period. The purity of samples was detected by a Spectrophotometer (NanoPhotometer^®^IMPLEN, CA, USA). 2100 RNA Nano 6000 Assay Kit (Agilent Technologies, CA, USA) was used for detecting RNA integrality as quality control. cDNAs of Reverse Transcription were sequenced Novaseq 6000 S4 reagent kit v1.5 on a Novaseq 6000 S4 platform. Significant DEGs were screened using read count data of the gene expression in each sample obtained by expression quantification with DESeq2 software. Fold changes in the expression levels between samples were used as the criteria in the screening process. After merging the DEGs from two treatments, clustering analyses were performed using Cluster 3.0 and Java TreeView. DAVID tool and Metascape tool were applied to the analysis and visualization of differentially expressed genes.

### siRNA transient transfection and plasmid transfection

Cells were plated and cultured to 60% confluence and transfected for 24 h with 100 nM ALOX15-targeted siRNA or negative control siRNA using Lipofectamine 2000 Reagent (Invitrogen) following the manufacturer’s protocol. siRNAs were purchased from GenePharma (Shanghai, China). The sequences for *Alox15* were GGAUGUGUCAGGAUACCUUTT (sense), AAGGUAUCCUGACACAUCCTT (anti-sense); CCAGAAAGGCACUCUGUUUTT (sense), AAACAGAGUGCCUUUCUGGTT (antisense); GCUUUACACCAUGGAAAUUTT (sense); AAUUUCCAUGGUGUAAAGCTT (anti-sense). The sequences of negative control were UUCUCCGAACGUGUCACGUTT (sense), ACGUGACACGUUCGGAGAATT(anti-sense). Western blot was used to verify the effect of gene silencing. A combination of three siRNA sequences (1:1:1) was applied in these experiments.

For ALOX15 overexpression, cells were plated in a 6-well plate at a density of 4.0 × 10^6^ and transfected with pAAV-CMV-*Alox15*-3xFlag or empty vector (VectorBuilder, Guangzhou, China) using NEOFECT DNA transfection reagent (Beijing, China). Six hours later, the complete medium was changed for another 24 h culture. Overexpression of ALOX15 was confirmed by western blot analysis.

### Western blot analysis

Protein extracts from cell or animal samples were prepared on ice by RIPA buffer (Beyotime, Shanghai China) containing protease and phosphatase inhibitors. The protein lysates were separated by sodium dodecyl sulfate-polyacrylamide gel electrophoresis and transferred to 0.45 μm or 0.22 μm PVDF membrane (Merck Millipore, Billerica, MA, USA). The transferred proteins were incubated with primary antibodies against ALOX15 (ab244205, Abcam), Bax (2772 S, Cell Signaling Technology), Bcl2 (2870, Cell Signaling Technology), caspase-1 (SC-56036, Santa Cruz) and GAPDH (FD0068, Fdbio Science, Hangzhou, China), and followed by incubation with horseradish peroxidase (HPR) conjugated secondary antibodies (FDR007/FDM007, Fdbio science, Hangzhou, China). After probed by ECL kit (Fdbio science, Hangzhou, China), the protein expression level was visualized under Tanon 5200 Chemiluminescence Image Analysis System (Tanon Science & Technology, Shanghai, China). The semi-quantifications of protein expressions were analyzed using ImageJ (NIH, USA).

### Quantitative real-time PCR analysis

TRIzol (Invitrogen) was used to extract total RNA from heart tissues or cell samples according to the manufacturer’s protocol. RNA was reversely transcribed into cDNA using TransScript^®^ One-Step gDNA Removal and cDNA Synthesis SuperMix (Transgen, Beijing, China). TransStart® Top Green qPCR SuperMix (Transgen, Beijing, China) was used to perform quantitative PCR according to the manufacturer’s instructions. The signals were detected and analyzed on CFX Connect System (Bio-rad, USA). GAPDH or β-actin was used as a standard control, and primer sequences were listed in Supplementary Table 1.

### Measurement of AST, LDH and CK-MB levels

Myocardial enzymes AST, LDH, and CK-MB had been regarded as clinically diagnostic values. Accordingly, blood was collected from mice into EP tubes by an eye enucleation method. Blood in the EP tube was centrifuged at 3000 rpm for 15 min to serum. Myocardial enzymes in the serum were then determined using Chemistry Analyzer AU680 (Beckman coulter, USA). The whole blood viscosity was analyzed by an automatic blood rheometer LBY-N7500B (Precil, Beijing, China).

### MTT assay

Cells were distributed in 96-well plates with a density of 5 × 10^3^. After treatments, 20 μL of 5 mg/ml MTT solution was added to cells and incubated for 4 h. The produced formazan was dissolved into 150 μL DMSO and detected by a multi-functional microplate system (Multiskan MK3, Thermo Fisher Scientific) at an absorbance of 490 nm.

### LDH/ SOD/MDA/NADPH detection

Cells were distributed in 6-well plates with a density of 10 × 10^6^. After treatments, culture supernatant was collected to detect LDH release using a commercially available kit. For SOD/MDA/NADPH measurement, cells were lysed by the repetitive freeze-thaw method and the supernatant was used to determine the activity of SOD and the levels of MDA and NADPH by commercial kits. All the assays were strictly conducted in the guidance of kit manuals. The LDH, SOD and MDA kits were purchased from Beyotime Co., Ltd. (Shanghai, China), and the NADPH kit was from Comin Biotechnology Co., Ltd. (Suzhou, China).

### The determination of glutathione content

GSH in the heart tissues (10 mg) was prepared by a methanol-water extraction method as previously described.^63^ Briefly, ultrasonicated solutions of heart tissues were collected with an equal proportion of methanol and water at 4 °C. The solution was extracted using 500 μL dichloromethane, and the retained upper liquid centrifuged at 14000 rpm for 25 min at 4 °C. After drying using nitrogen, the samples were dissolved in 200 μL of 50% methanol-water, centrifuged at 14000 rpm for 25 min under 4 °C again, and analyzed by HPLC-MS (Thermo Fisher Scientific).

### Cellular Oil Red O staining

Oil Red O staining was performed to detect lipid accumulation in PUFA-enriched and MUFA-enriched cells. After treatment, cells were washed twice with PBS and fixed with reagent A in Oil Red O Kit (Shanghai Yuanye Bio-Technology Co., Ltd., Shanghai, China) at room temperature for 20 min, and then was stained with reagent B for 15 min. Next, cells were rinsed with 60% isopropanol and nuclei were counterstained with reagent C for 2 min. After washing with reagent D for 1 min, the images of stained cells were acquired by an IX51 inverted microscope (Olympus, Tokyo, Japan).

### Oxygen radical absorbance capacity (ORAC) analysis

The free radical scavenging capacity of daidzein (Chroma-Biotechnology Co., Ltd., Chengdu, China) was tested by an ORAC method as previously described.^64^ In brief, 2,2’-Azobis(2-methylpropionamidine) dihydrochloride (AAPH, a free radical generator), fluorescence sodium (Sigma, MO, USA, fluorescence probe excitation/emission at 485/535 nm), and Trolox (free radical scavenger) were used. In this reaction system, 20 μL of 75 mM PBS, 20 μL of daidzein, 20 uL of fluorescein and 140 μL of AAPH were included. The fluorescence was measured every 2 min for 2 h using a TECAN GENios Luciferase microplate reader (Männedorf, Switzerland) at 37 °C. The value of ORAC was calculated as the net area under the fluorescence decay curve using Trolox as the calibration standard.

### ALOX15 inhibitor screen by molecular docking

A total 40271 molecules from the Natural Compound Library of Topscience and MedChemExpress were docked with ALOX15 (PDB ID: 1LOX) by libdock module of discovery Studio software (version 3.5). Flexible docking of 10000-selected matching molecules (score greater than 120) to each potential binding site on ALOX15 was performed using the CDOCKER module, acquiring 212 matching compounds (score greater than 25). Finally, based on structural clustering and binding pattern analysis, daidzein was selected as a potential ALOX15 inhibitor with the best score.

### CETSA

CETSA was performed in the lysates of ALOX15-overexpressing HEK293T cells. After transfection for 24 h, cells were collected and freeze-thawed under liquid nitrogen, and cell lysates were diluted with PBS and divided into two aliquots, with one aliquot being added with daidzein (50 μM) and the other as a DMSO control. After incubation at 37 °C for 1 h, all lysates were heated at different temperatures from 41 to 73 °C for 3 min, followed by cooling at room temperature. All the samples were centrifuged at 4 °C and the soluble fractions were subjected to western blot analysis.

### MST

Daidzein was diluted with MST buffer (50 mM HEPES, 150 mM NaCl, 10 mM MgCl2, 0.05% Tween-20, pH 7.0) to 1 mM. Lipoxygenase from soybean (L7395, Sigma, China) was labeled by a Monolith NT^TM^ RED-NHS Second Generation Protein Labeling Kit based on the manual (NanoTemper Technologies GmbH, Munich Germany) and diluted with MST buffer to 100 nM. The mixture of daidzein and labeled-ALOX15 was incubated at room temperature for 30 min and their binding affinity was analyzed by microscale thermophoresis (NanoTemper Monolith Instrument NT.115). Red-laser was absorbed by mixed aqueous solutions of different concentrations in 12 capillaries. Fluorescence was determined in a thermal gradient at 40% MST power with laser off/on times of 5–20 s, and data was analyzed by MO Affinity Analysis v2.3.

### Statistical analysis

Data are presented as mean ± SD. Where applicable, normality was estimated using Levene’s test. *P* > 0.05 was considered to conform to the normal distribution. For comparison between 2 groups, independent samples *t* test was used when data presented normal distribution and Mann–Withney test was used when data did not follow normal distribution. For more than 2 groups, the normal distribution data were analyzed by One-Way ANOVA with Turkey multiple comparison, and the non-normal distribution data were analyzed by Post Hoc Test with Dunnentt’s T3. (SigmaStat, SPSS Inc., Chicago, Illinois, USA). Comparison of survival curves was statistically analyzed by Log-rank (Mantel-Cox) test. *P* < 0.05 was considered statistically significant.

## Supplementary information

Revised Supplementary materials-unmarked

## Data Availability

The authors declare that the data supporting the findings of this study are available within the paper and its Supplementary materials. All data generated during this study are included in this published article and its supplementary information files. All data in this study are available from the corresponding author with a reasonable request.
