# Supplementary material for: ALOX15-launched PUFA-phospholipids peroxidation increases the susceptibility of ferroptosis in ischemia-induced myocardial damage
Source: Signal Transduct Target Ther. 2022 Aug 15;7:288. doi: 10.1038/s41392-022-01090-z (PMC9378747; doi:10.1038/s41392-022-01090-z)
Supplement: Supplementary file 1 — Revised Supplementary materials-unmarked [file 41392_2022_1090_MOESM1_ESM.docx]

Supplementary Materials for

**ALOX15-launched PUFA-phospholipids peroxidation increases the susceptibility of ferroptosis in ischemia-induced myocardial damage**

Xiao-Hui Ma^1-3,7^, Jiang-Han-Zi Liu^1-3^, Chun-Yu Liu^1-3^, Wan-Yang Sun^1-3^, Wen-Jun Duan^1-3^, Wang Guan^6^, Hiroshi Kurihara^1-3^, Yi-Fang Li^1-3*^, Yang Chen^4*^, Hong-cai Shang^5*^, Rong-Rong He^1-3*^

^1^Guangdong Engineering Research Center of Chinese Medicine & Disease Susceptibility, Jinan University, Guangzhou 510632, China; ^2^Guangdong Province Key Laboratory of Pharmacodynamic Constituents of TCM and New Drugs Research, College of Pharmacy, Jinan University, Guangzhou 510632, China; ^3^International Cooperative Laboratory of Traditional Chinese Medicine Modernization and Innovative Drug Development of Chinese Ministry of Education (MOE), Jinan University, Guangzhou 510632, China; ^4^College of Pharmacy, Guangzhou University of Chinese Medicine, Guangzhou 510405, China; ^5^Key Laboratory of Chinese Internal Medicine of Ministry of Education, Dongzhimen Hospital, Beijing University of Chinese Medicine, Beijing 100700, China; ^6^Innovation Center of Nursing Research, Nursing Key Laboratory of Sichuan Province, State Key Laboratory of Biotherapy and Cancer Center, West China Hospital, Sichuan University, Chengdu 610041, China. ^7^Institute of Traditional Chinese Medicine, Xinjiang Medical University, Urumqi 830054, China).

Correspondence to: rongronghe@jnu.edu.cn

**This PDF file includes:**

Figures. S1 to S8

Tables S1

**Supplementary Figure captions and legends**


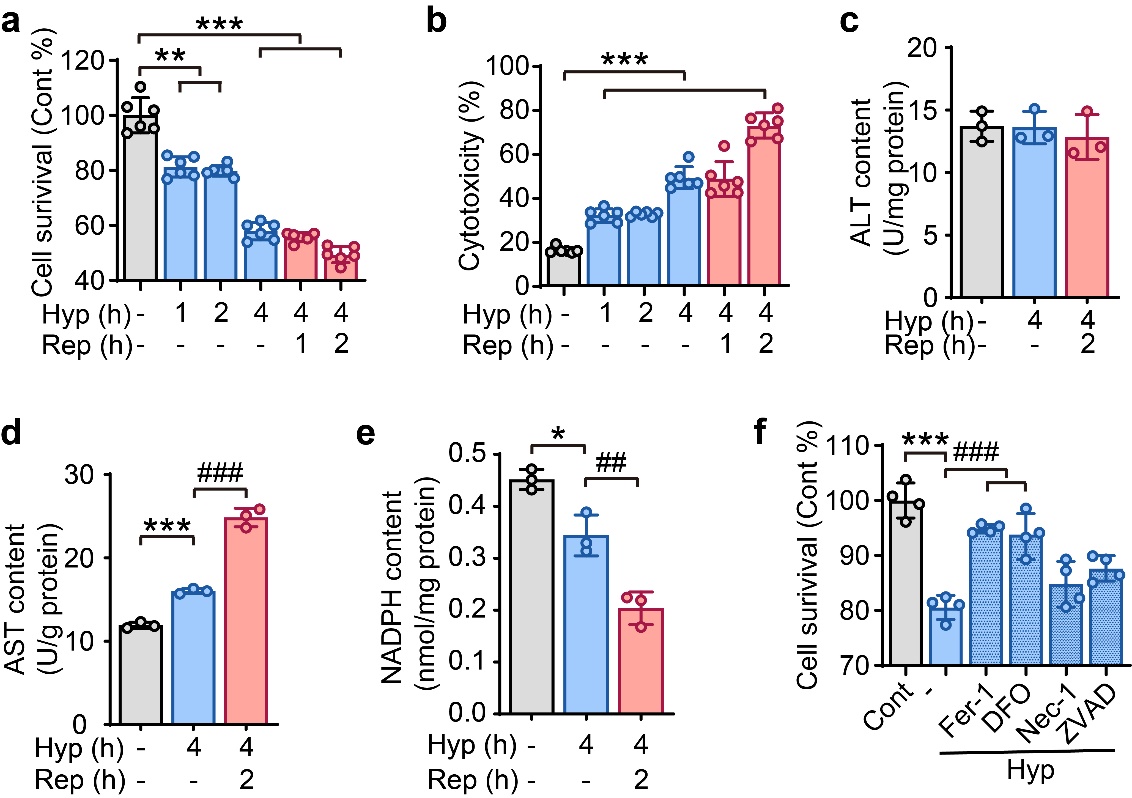


**Supplementary Fig. 1. Hypoxia causes cell damage and dysfunction in cardiomyocytes**

**a and b** Cytotoxicity caused by hypoxia/reoxygenation for different time periods in H9C2 cells. (**a**) MTT assay; (**b**) LDH measurement.

**c-e** Cell dysfunction induced by 4-hour hypoxia and/or 2-hour reoxygenation in H9C2 cells. (**c**) ALT level; (**d**) AST level; (**e**) NADPH content.

(**f**) Effect of different cell death inhibitors on cell survival in hypoxia H9C2 cells. ferroptosis inhibitor: ferrostatin-1 (Fer-1, 1 μM) and deferoxamine (DFO, 100 μM); necroptosis inhibitor: necrostatin-1 (Nec-1, 1 μM); apoptosis inhibitor: Z-VAD-FMK (ZVAD, 1 μM)

Data is expressed as mean ± SD, and statistical significance was analyzed by one-way ANOVA followed by the Tukey post-hoc test. ^*^*p* < 0.05, ^**^*p* < 0.01, ^***^*p* < 0.001 vs control (Cont) group; ^#^*p* < 0.05, ^##^*p* < 0.01, ^###^*p* < 0.001 vs the hypoxia (Hyp) group.


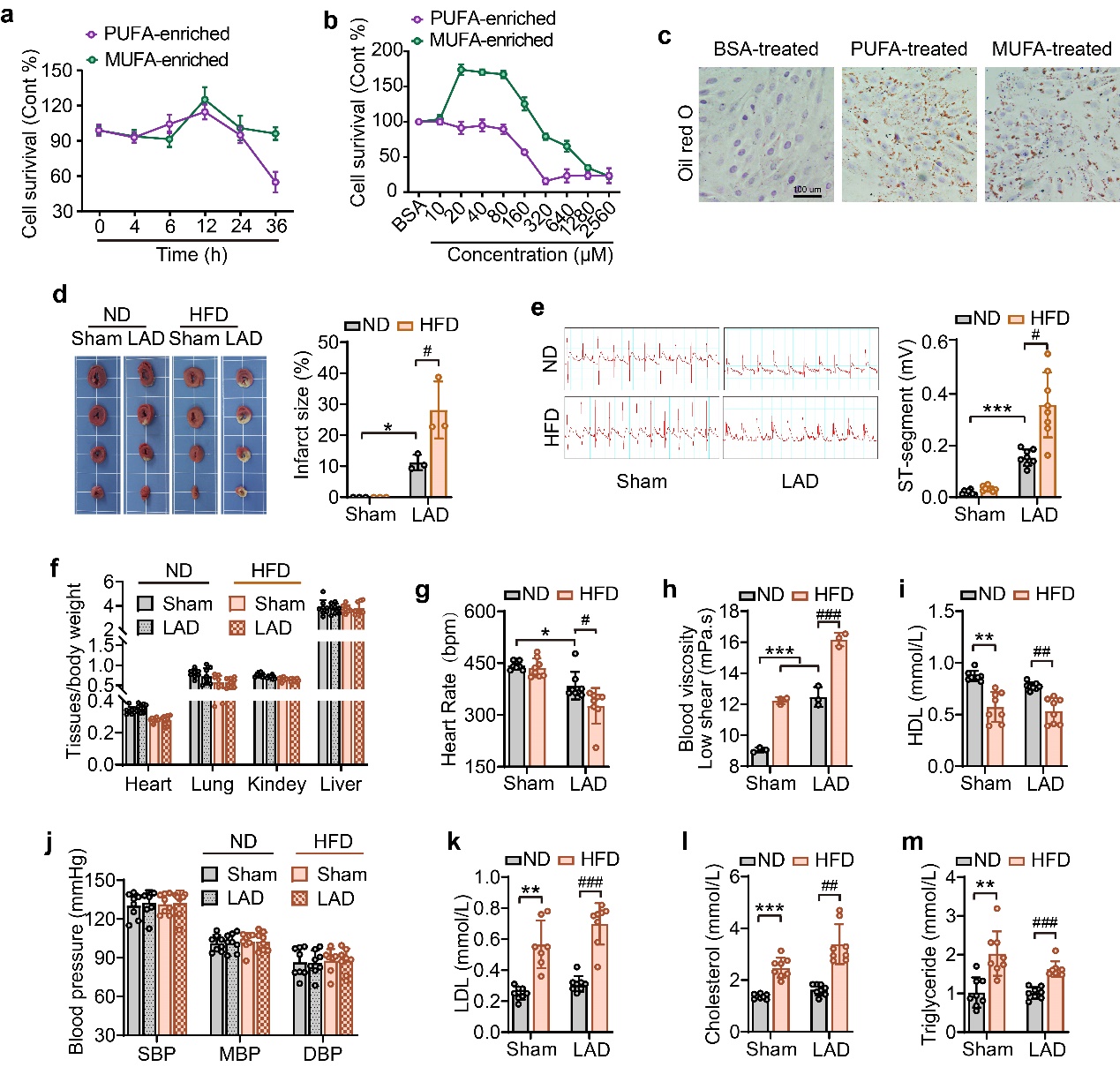


**Supplementary Fig. 2. The preparation of PUFA-enriched cells and the establishment of ischemia model by LAD ligation in PUFA-enriched rats.**

**a** Effect of OA (80 μM) or LA (80 μM) supplement on the survival of H9C2 cells at different time points.

**b** Effect of different concentrations of OA or LA supplement for 12 hours on the survival of H9C2 cells.

**c** Oil red O staining in LA or OA challenged cells. The coating agent BSA was set as the corresponding control.

**d** TTC staining of heart tissue sections from LAD-ligation rats fed with ND or PUFA-containing HFD. The quantitative analysis of infarct area was shown in the right panel (n = 3). Unstained white tissue represents ischemic areas, while stained red tissue is non-infarcted. The infarct size was analyzedusing ImageJ and expressed as the percentage of infarcted area relative to the whole heart area.

**e** Representative diagram of electrocardiogram (left panel) and statistics analysis of ST-segment elevation (right panel). ST-segment of ECG elevated over 0.1mV from baseline was regarded as an index of ischemia.

**f** The ratios of organ weight to body weight.

**g-m** The changes of cardiovascular function-related index. (**g**) Heart rate; (**h**) Blood viscosity; (**i**) high-density lipoprotein (HDL); (**j**) blood pressure; (**k**) low-density lipoprotein (LDL); (**l**) Cholesterol; (**m**) Triglyceride.

Data is expressed as mean ± SD, and statistical significance was analyzed by one-way ANOVA followed by the Tukey post-hoc test. ^*^*p* < 0.05, ^**^*p* < 0.01, ^***^*p* < 0.001 vs “ND+sham” group; ^#^*p* < 0.05, ^##^*p* < 0.01, ^###^*p* < 0.001 vs “ND+LAD” group.


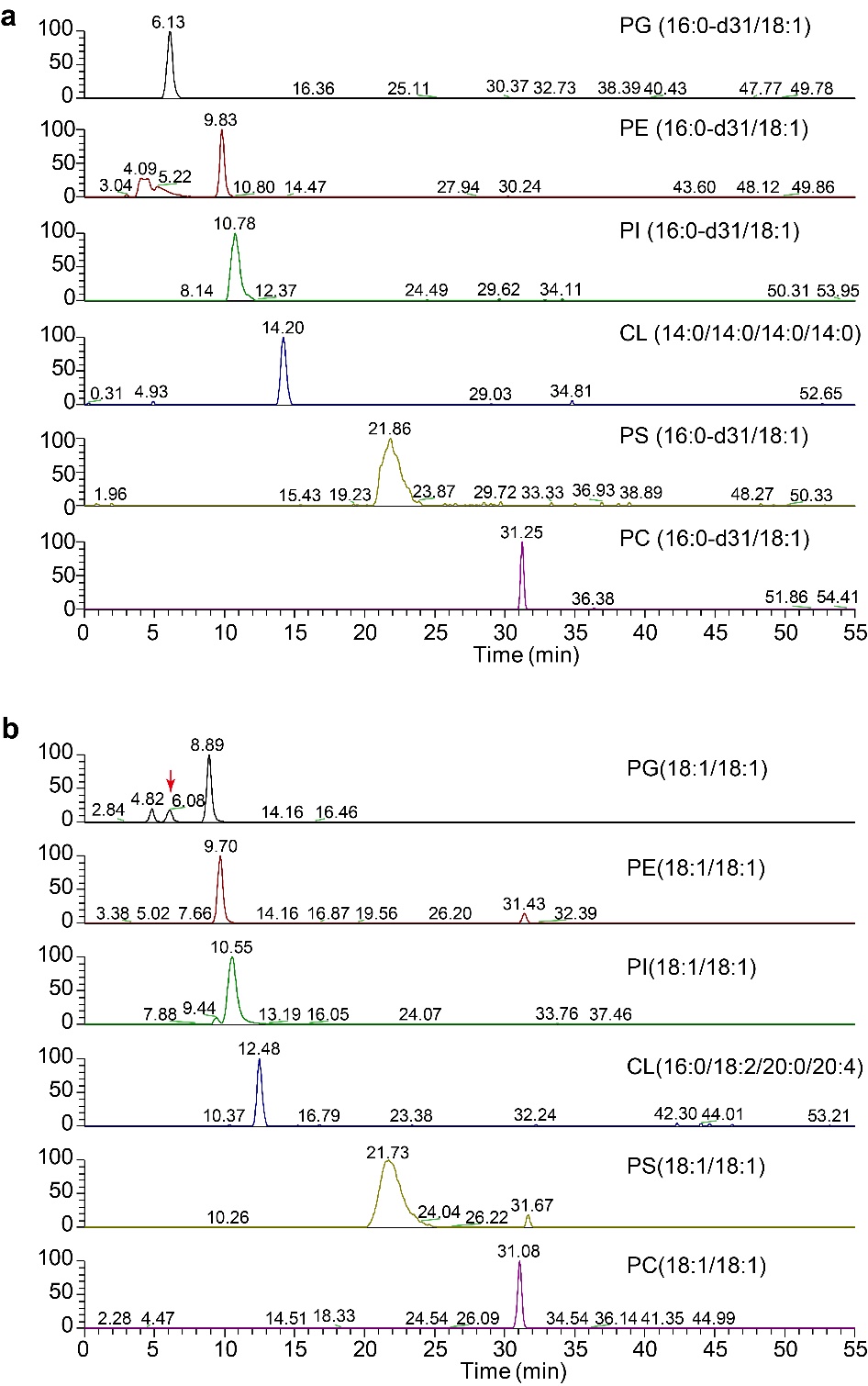


**Supplementary Fig. 3. The extracted ion chromatogram of internal standard substances (a) and phospholipid reference standards (b).** Extracted ion currents of six phospholipid reference standards and six internal standard substances are shown above. In normal phase system, phospholipids were separated by their head groups. The retention time of each reference standard could be used to help distinguish different classes of phospholipids.


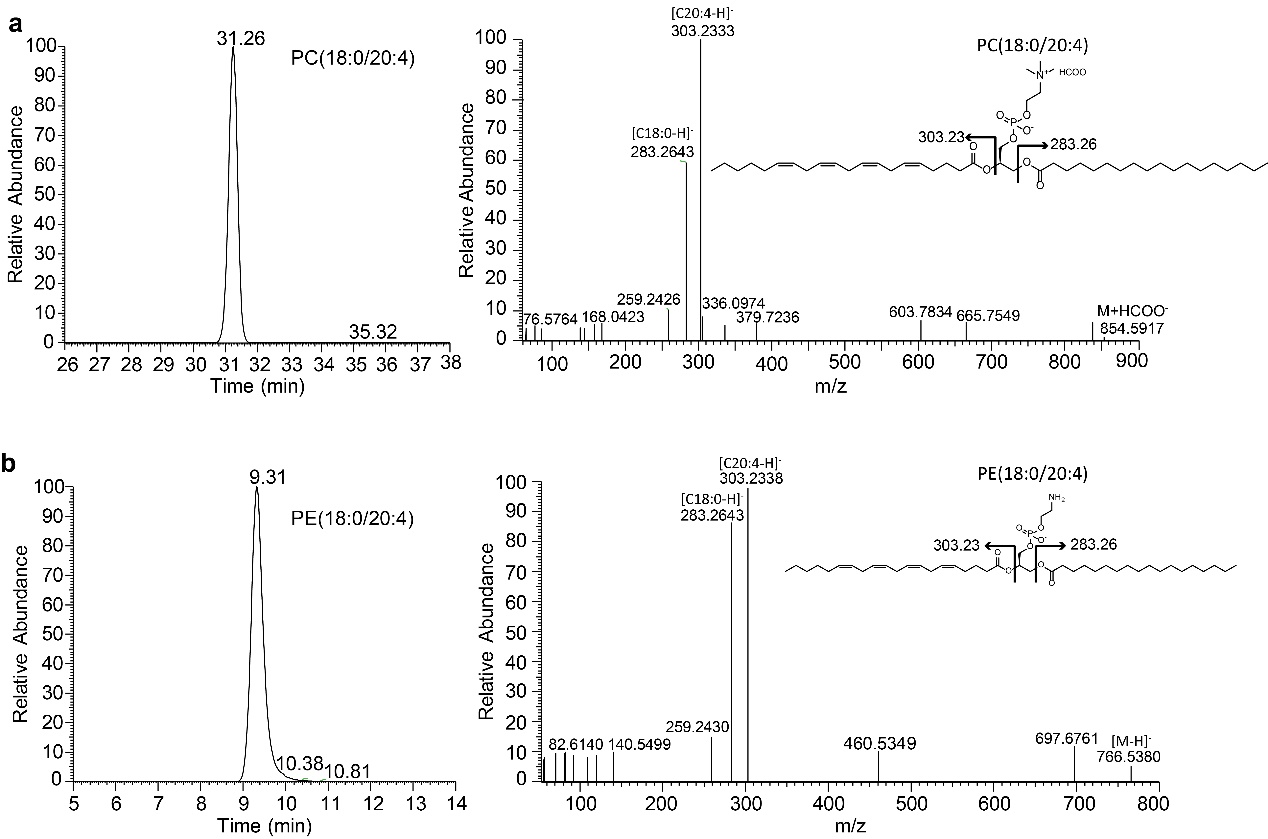


**Supplementary Fig. 4. The identification of phospholipids by A secondary mass spectrometer.**

**a** The extracted ion current of PC (16:0/18:1) (the right panel) and its characteristics fragments by MS/MS spectrum (the left panel). The full scan mass spectrum gave a [M+HCOO]^-^ ion at *m/z* 804.5754, corresponding to the molecular formula (C_43_H_84_O_10_NP) of PC (34:1). The MS^2^ spectrum of *m/z* 804.5754 shows a minor carboxylate anion at *m/z* 255.2325 ([16:0-H]^-^), a major carboxylate anion fragment at *m/z* 281.2486 ([18:1-H]^-^), and a [H-H-R_2_CH=C=O-CH_2_]^-^ fragment at *m/z* 480.3105 due to the loss of the *sn*-2 acyl chain as ketene (R_2_CH=C=O), CH_2_ and formate moieties from precursor ion. Collectively, it was assigned as PC (16:0/18:1).

**b** The extracted ion current of PE (18:0/20:4) (the right panel) and its characteristics fragments by MS/MS spectrum (the left panel). The full scan mass spectrum gave a [M-H]^-^ ion at *m/z* 766.5387, corresponding to the molecular formula (C_43_H_78_O_8_NP) of PE (38:4). The MS^2^ spectrum of *m/z* 766.5387 shows a minor carboxylate anion at *m/z* 283.2644 ([18:0-H]^-^), a major carboxylate anion fragment at *m/z* 303.2331 ([20:4-H]^-^), and a [H-H-R_2_CH=C=O-CH_2_]^-^ fragment at *m/z* 480.3105 due to the loss of the *sn*-2 acyl chain as ketene (R_2_CH=C=O) from precursor ion. Collectively, it was assigned as PE (18:0/20:4).


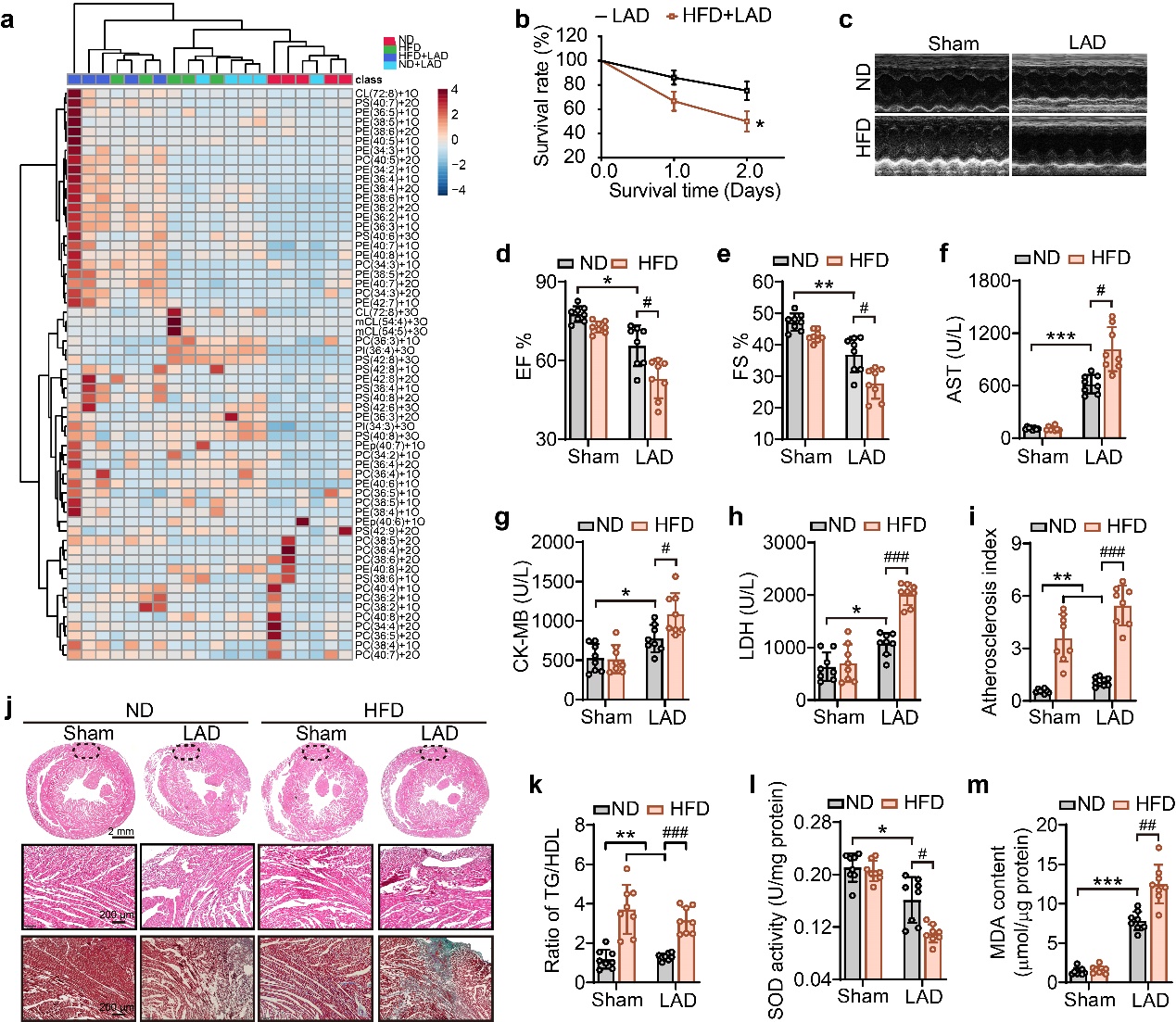


**Supplementary Fig. 5. PUFA-enriched HFD feeding aggravates heart dysfunction induced by LAD ligation ischemia in rats**

**a** Heat map of different oxidized phospholipids in sham or LAD-ligation rats fed with ND or PUFA-enriched HFD (n=5).

**b** Influence of PUFA-enriched HFD on the survival rate of rats subjected to LAD surgery (n = 36). ^*^*p* < 0.05 by Log-rank (Mantel-Cox) test.

**c-e** Representative echocardiography images for ND- or HFD-fed rats subjected to LAD-ligation (**c**); Quantitative analysis of left ventricular ejection fraction (EF%) (**d**) and left ventricular shortening fraction (FS%) (**e**).

**f-h** Effect of PUFA-enriched HFD on the contents of myocardial enzymes, including AST (**f**), CK-MB (**g**) and LDH (**h**) in LAD-ligation rats.

**i and k** Effect of PUFA-enriched HFD on coronary heart disease-related parameters, including Atherosclerosis index (**i**) Atherosclerosis index was calculated as the formula: AI=(TC-HDL)/HDL and (**k**) Ratio of TG/HDL.

**j** H&E and Masson staining of heart tissue sections (n = 3).

**l and m** MDA content (**l**) and SOD activity (**m**) of heart tissues of rats.

Data is expressed as mean ± SD, and statistical significance was analyzed by one-way ANOVA followed by the Tukey post-hoc test. ^*^*p* < 0.05, ^**^*p* < 0.01, ^***^*p* < 0.001 vs “ND+sham” group; ^#^*p* < 0.05, ^##^*p* < 0.01, ^###^*p* < 0.001 vs “ND +LAD” group.


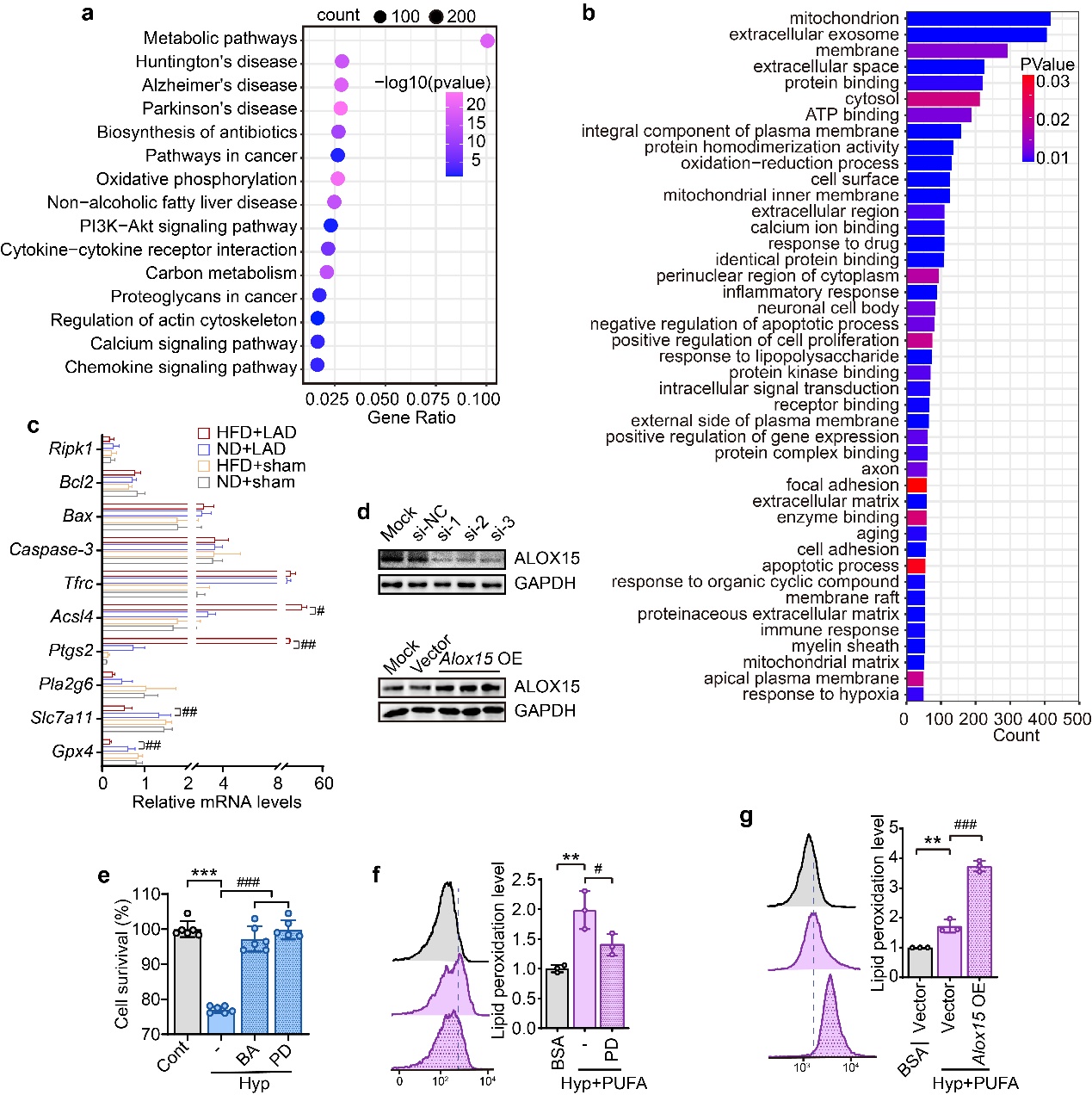


**Supplementary Fig. 6.** **ALOX15 is responsible for PUFA-induced susceptibility to myocardial** **ischemia**

**a** KEGG analysis enriched top 15 alteration pathways by between “ND+sham” and “ND+LAD” groups. Gene ratio refers to the genes enriched in entries. The color of the circle represents the -log_10_ (*p*-value), and the size of the circle relates to the number of genes enriched in the duplicate entry (n=4).

**b** GO analysis enriched disturbed biological process category between “ND+sham” and “ND+LAD” groups. The color of the circle represents the *p*-value (n=4).

**c** A quantitative RT-PCR analysis of genes related with ferroptosis (*Acsl4*, *Ptgs2*, *Tfrc*, *Slc7a11*, *Gpx4,* and *Pla2g6*), apoptosis (*Bcl_2_*, *Bax,* and *caspase-3*), and necrosis (*Ripk1*) in heart tissues of LAD-ligation rats fed with ND or HFD (n=3).

**d** The verification of ALOX15 protein expression by Western blot in H9C2 cells treated with siRNA or overexpression (n=3).

**e** Effect of ALOX15 inhibitors, including baicalein (BA) and PD 146176 (PD), on cell survival in hypoxia H9C2 cells. BA (50 μM) and PD (10 μM) were pretreated for 2 hours before hypoxia treatment. Data is expressed as mean ± SD, and statistical significance was analyzed by one-way ANOVA followed by the Tukey post-hoc test. ^***^*p* < 0.001 vs control (Cont) group; ^###^*p* < 0.001 vs hypoxia (Hyp) group.

**f** and **g** Effect of PD 146176 (**f**) or *Alox15* overexpression (**g**) on the accumulation of lipid ROS were detected by Liperfluo staining in PUFA-enriched H9C2 cells exposed to hypoxia. Data is expressed as mean ± SD, and statistical significance was analyzed by one-way ANOVA followed by the Tukey post-hoc test. ^**^*p* < 0.01, vs control (Cont) group; ^#^*p* < 0.05, ^###^*p* < 0.001 vs “Hyp+PUFA” group.


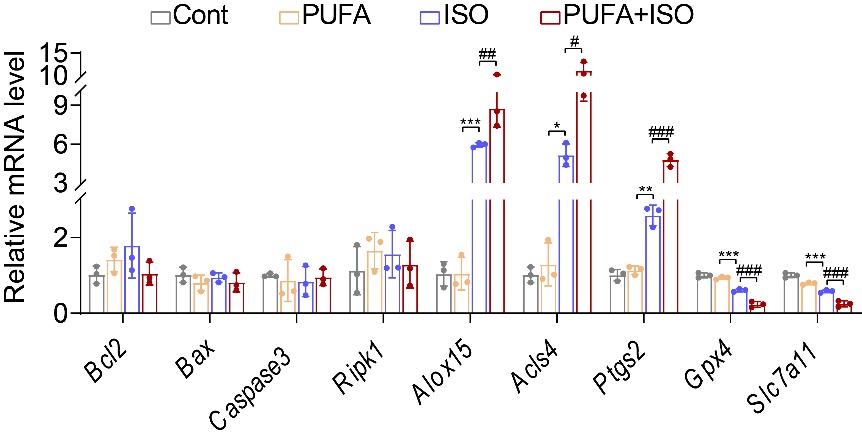


**Supplementary Fig. 7.**  **A quantitative RT-PCR analysis of genes related with ferroptosis, apoptosis and necrosis in ISO-induced ischemic heart tissues of PUFA-enriched mice**. Data is expressed as mean ± SD (n=3), and statistical significance was analyzed by one-way ANOVA followed by the Tukey post-hoc test. ^*^*p* < 0.05, ^**^*p* < 0.01, ^***^*p* < 0.001 vs PUFA group; ^#^*p* < 0.05, ^##^*p* < 0.01, ^###^*p* < 0.001 vs “PUFA+ISO” group.


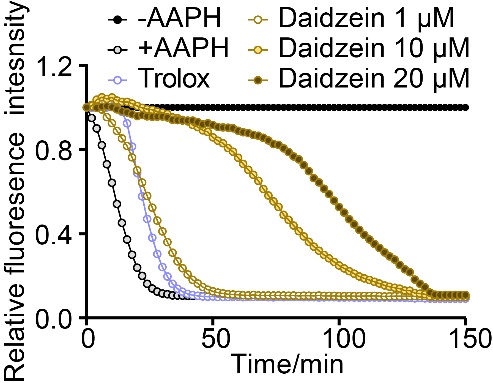


**Supplementary Fig. 8.**  **Direct ROS scavenging capacity of daidzein was evaluated by in vitro oxygen radical absorbance capacity (ORAC) assay**

AAPH (2,2'-Azobis(2-methylpropionamidine) dihydrochloride) and fluorescence sodium were, respectively, used as a free radical generator as a fluorescence probe (excitation/emission at 485/535 nm). Trolox (a water soluble vitamin E analog) was as a standard radical scavenger. The value of ORAC was calculated as the net area under the fluorescence decay curve using Trolox as the calibration standard.

**Supplementary Table 1. Primer information**

| gene | sequence (5'->3') |
| --- | --- |
| *Gpx4* | F: GACCTTCCCCAGACCAGCAAC  R: ACGCAACCCCTGTACTTATCC |
| *Slc7a11* | F: TCGGATCGGGCATCTTCATC  R: CGGGGCGTATTACCAGCAGT |
| *Pla2g6* | F: CAATGGACGCTTCTTGGATGG  R: ACAGGCACTTGAGGGGACTTT |
| *Alox15* | F: CATGTTCCCCTGTTACCGAT  R: GCTCCTCTTCCCTATGTTTCC |
| *Alox12* | F: CCTCTACGCTCAGGATGCTTT  R: CTGGGTCTCCCCTCACAATAT |
| *Alox5* | F: GAGTTCCCTTGTTATCGTTGG  R: CTTGAGGATGTGGATTTGGTC |
| *Acsl4* | F: CCATATCGCTCTGTCACGCACTT  R: CCCCAGGCTGTCCTTCTTCC |
| *Ptgs2* | F: TCAATGAGTACCGCAAACGC  R: TGGTCTCCCCAAAGATAGCA |
| *Ripk1* | F: AACTCCAGTGCCTGAGACCA  R: TTAGCGAAGACGGCTTGATG |
| *Bcl2* | F: ACTTCTCTCGTCGCTACCGTCG  R: CCCTGAAGAGTTCCTCCACCACC |
| *Bax* | F: CATGAAGACAGGGGCCTTTTTG  R: TCAGCTTCTTGGTGGATGCGTC |
| *Caspase3* | F: GCAGTTTTGTGTGTGTGATT  R: GAGTTTCGGCTTTCCAGT |
| *Gapdh* | F: GCTCTCTGCTCCTCCCTGTTCTA  R: AATCCGTTCACACCGACCTTCA |
